# Health-Related Quality of Life in SCALOP, a Randomized Phase 2 Trial Comparing Chemoradiation Therapy Regimens in Locally Advanced Pancreatic Cancer

**DOI:** 10.1016/j.ijrobp.2015.08.026

**Published:** 2015-11-15

**Authors:** Christopher N. Hurt, Somnath Mukherjee, John Bridgewater, Stephen Falk, Tom Crosby, Alec McDonald, George Joseph, John Staffurth, Ross A. Abrams, Jane M. Blazeby, Sarah Bridges, Peter Dutton, Gareth Griffiths, Tim Maughan, Colin Johnson

**Affiliations:** ∗ Wales Cancer Trials Unit, College of Biomedical and Life Sciences, Cardiff University, Cardiff, Wales, United Kingdom; † Cancer Research UK/MRC Oxford Institute for Radiation Oncology, Oxford University, NIHR Biomedical Research, Oxford, United Kingdom; ‡ UCL Cancer Institute, London, United Kingdom; § Bristol Haematology and Oncology Centre, Bristol, United Kingdom; ‖ Velindre Cancer Centre, Velindre Hospital, Cardiff, Wales, United Kingdom; ¶ Beatson West of Scotland Cancer Centre, Glasgow, Scotland, United Kingdom; # Institute of Cancer and Genetics, Cardiff University, Cardiff, Wales, United Kingdom; ∗∗ Department of Radiation Oncology, Rush University Medical Center, Chicago, Illinois; †† Division of Surgery, Head and Neck, University Hospitals Bristol National Health Service Foundation Trust, Bristol and School of Social and Community Medicine, University of Bristol, Bristol, United Kingdom; ‡‡ Centre for Statistics in Medicine, University of Oxford, Oxford, United Kingdom; §§ Southampton Clinical Trials Unit, Faculty of Medicine, Southampton University, Southampton General Hospital, Southampton, United Kingdom; ‖‖ University Surgical Unit, Faculty of Medicine, University Hospital Southampton, Southampton, United Kingdom

## Abstract

**Purpose:**

Chemoradiation therapy (CRT) for patients with locally advanced pancreatic cancer (LAPC) provides survival benefits but may result in considerable toxicity. Health-related quality of life (HRQL) measurements during CRT have not been widely reported. This paper reports HRQL data from the Selective Chemoradiation in Advanced Localised Pancreatic Cancer (SCALOP) trial, including validation of the QLQ-PAN26 tool in CRT.

**Methods and Materials:**

Patients with locally advanced, inoperable, nonmetastatic carcinoma of the pancreas were eligible. Following 12 weeks of induction gemcitabine plus capecitabine (GEMCAP) chemotherapy, patients with stable and responding disease were randomized to a further cycle of GEMCAP followed by capecitabine- or gemcitabine-based CRT. HRQL was assessed with the European Organization for Research and Treatment of Cancer Quality of Life Questionnaire (EORTC QLQ-C30) and the EORTC Pancreatic Cancer module (PAN26).

**Results:**

A total of 114 patients from 28 UK centers were registered and 74 patients randomized. There was improvement in the majority of HRQL scales during induction chemotherapy. Patients with significant deterioration in fatigue, appetite loss, and gastrointestinal symptoms during CRT recovered within 3 weeks following CRT. Differences in changes in HRQL scores between trial arms rarely reached statistical significance; however, where they did, they favored capecitabine therapy. PAN26 scales had good internal consistency and were able to distinguish between subgroups of patients experiencing toxicity.

**Conclusions:**

Although there is deterioration in HRQL following CRT, this resolves within 3 weeks. HRQL data support the use of capecitabine- over gemcitabine-based chemoradiation. The QLQ-PAN26 is a reliable and valid tool for use in patients receiving CRT.

**Summary**

The Selective Chemoradiation in Advanced Localised Pancreatic Cancer trial was a randomized, phase 2 trial in which patients with locally advanced, inoperable pancreatic cancer were given capecitabine- or gemcitabine-based chemoradiation. This paper reports the health-related quality of life (HRQL) data, including validation of the QLQ-PAN26 tool in chemoradiation therapy. Data support the use of chemoradiation as a treatment option (with capecitabine-based chemoradiation preferred) and the use of the QLQ-PAN26 as a valid tool.

## Introduction

Pancreatic cancer patients have a 5-year survival rate of less than 5% (1). Treatment with chemoradiation therapy (CRT) may improve overall survival in patients with locally advanced inoperable tumors but may result in considerable toxicity (2). Health-related quality of life (HRQL) measurements, not widely reported in the published reports, are therefore relevant when interpreting trial data and when making treatment recommendations for patients with advanced pancreatic cancer.

The Selective Chemoradiation in Advanced Localised Pancreatic Cancer (SCALOP) trial was a randomized phase 2 trial that compared gemcitabine-based CRT (Gem-CRT) and capecitabine-based CRT (Cap-CRT) following a course of induction chemotherapy in locally advanced pancreatic cancer (LAPC). The SCALOP trial demonstrated that Gem-CRT was associated with more instances of Common Terminology Criteria for Adverse Events (CTCAE) grade 3 and 4 hematological and nonhematological toxicities and inferior median survival (Gem-CRT 13.4 vs Cap-CRT 15.2 months, *P*=.012) (3). In SCALOP, HRQL was assessed with the European Organization for Research and Treatment of Cancer (EORTC) QLQ-C30 (4) and the pancreatic cancer module EORTC QLQ-PAN26 (5). The PAN26 was developed for patients undergoing surgery, palliative chemotherapy, and endoscopic treatment of pancreatic cancer, but has not been previously validated in CRT.

This paper describes generic, disease- and treatment-specific HRQL during and after treatment with CRT. It also provides validation and reliability data on QLQ-PAN26 in patients receiving CRT.

## Methods and Materials

### Participants and methods

SCALOP was a multicenter, open-label, randomized, parallel, 2-arm, phase 2 trial conducted in the United Kingdom (3). Patients with locally advanced, inoperable nonmetastatic, histologically confirmed carcinoma of the pancreas were eligible. Registered patients received 3 cycles of gemcitabine and capecitabine (GemCap) chemotherapy and were then restaged with CT scans of the thorax, abdomen, and pelvis. Patients with stable or responding disease (according to Response Evaluation Criteria In Solid Tumors criteria, version 1.1), a tumor diameter of 6 cm or less, and a World Health Organization performance status 0 to 1 were randomized 1:1 to either Gem-CRT or Cap-CRT by stratified minimization with a random element (80:20). All participants provided written informed consent. The study was approved by the UK Medical Research and Ethics Committee and UK Medicines and Healthcare products Regulatory Agency (MHRA). This study was registered at International Standard Randomised Controlled Trial Number (ISRCTN), number 96169987. The full protocol can be accessed at: http://www.wctu.org.uk/publications/scalop/SCALOP%20Clinical%20Protocol%20v4.0.pdf.

### Treatment protocol

Induction chemotherapy consisted of 3 cycles of gemcitabine (1000 mg/m^2^ intravenously over 1 hour on days 1, 8, and 15 of a 28-day cycle) and capecitabine (830 mg/m^2^ orally, twice daily on days 1 to 21 of a 28-day cycle). Randomized patients received a further cycle of GemCap followed by concurrent chemoradiation therapy in combination with either gemcitabine (300 mg/m^2^ once per week) or capecitabine (830 mg/m^2^ twice daily on days of radiation therapy only). The total radiation therapy dose was 50.4 Gy in 28 daily fractions over 5.5 weeks by use of 3-dimensional conformal or intensity modulated radiation therapy planning. No subsequent adjuvant therapy was given.

### Health-related quality of life

HRQL was assessed using the HRQL generic instrument, the EORTC QLQ-C-30, which assesses global quality of life, functional domains (physical, emotional, social, role, and cognitive) and symptoms (fatigue, nausea and vomiting, pain, dyspnea, insomnia, appetite loss, constipation, diarrhea, and financial difficulty) that commonly occur in patients with cancer (4), and a disease-specific measure, the EORTC QLQ-PAN26 (pancreatic domain, which uses 26 questions hypothesized as 17 scales and single items specifically related to pancreatic disease symptoms, treatment side-effects, and emotional issues) (5). Patients self-completed paper questionnaires at 6 time points: week 0 (baseline), week 17 (post-induction chemotherapy), week 23 (immediately post-CRT), and subsequently at follow-up (weeks 26, 39, and 52), even, where possible, if patients experienced disease progression. Questionnaires were included if completed within 1 week (4 weeks for weeks 39 and 52) of the specified time point. The EORTC standard scoring procedure is that function scales and items are defined such that higher scores represent better HRQL, whereas symptom scales and items are defined such that higher scores indicate more symptoms (worse HRQL). The full list is reported in Table 1.

### Data analysis

All randomized patients were included in the analysis. Analyses were prespecified in the statistical analysis plan and performed on an intention-to-treat basis (3). All analyses were undertaken and graphs produced using Stata version 13.0 software (Stata Corp., College Station, TX).

Data were imputed according to EORTC guidance if less than half the items within a scale were missing (6). Where data were missing from more than half the items within any scale, these scales were excluded from analyses. When a complete questionnaire was missing, the reason for the missing questionnaire was ascertained and categorized.

We performed 2 sets of analyses; 1 set investigated the change in HRQL during induction chemotherapy (weeks 0 to 17) and the other set analyzed the change from the start of CRT (week 17) and later time points to assess the specific impact of CRT on HRQL and difference between arms.

Changes in mean HRQL between earlier and later time points in all patients were normally distributed (assessed using Shapiro-Wilk tests for normality) and were presented with mean scores at each time point, changes in mean scores, and 95% confidence intervals around those changes. Changes in scores of 10 or more points were considered clinically significant (7). When these data were split by treatment arms to compare changes in HRQL during and after CRT, the data were no longer normally distributed, and therefore, Wilcoxon rank sum tests were used to compare changes between arms. We had no a priori hypotheses as to which specific scales would be most affected by which arm, so we compared all scales and highlighted results at a *P* level of <.05 (and a *P* level of <.01 to reduce errors from multiple testing) in these exploratory analyses.

### Psychometric testing of the QLQ-PAN26

Cronbach's alpha coefficient was calculated as a measurement of reliability of the QLQ-PAN26, using data from the week-23 assessments. Cronbach's alpha measures intercorrelation between the test scores of related items within the scales, and an alpha value of ≥0.70 indicates good consistency (8). Construct validity was assessed by observed differences in the scales at the time point immediately after CRT (week 23) between the group of patients who had any CTCAE grade 3 or 4 recorded by nurses and those who did not. It was hypothesized that patients with grade 3 or 4 adverse events would report worse scores in more scales than patients without any events. Additional known group comparisons were made in the “side effects scale” between patients with and without a serious adverse reaction (SAR) persisting at the week-23 time point, where symptoms are typically most severe. SARs were defined with at least the possibility of a causal relationship to one of the trial medications (including radiation therapy).

### Role of funding source

The study was funded by Cancer Research UK Clinical trials Awards and Advisory Committee (CRUK 07/040), which had no role in study design, data collection, analysis, or interpretation or writing of this report.

## Results

**Fig. 1:**
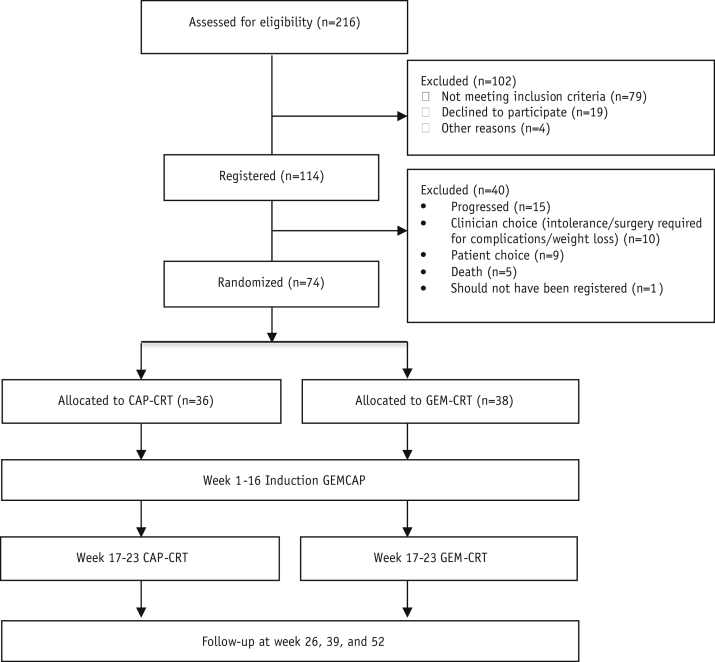
Flow diagram. *Abbreviations:* CAP_CRT = capecitabine-based chemoradiation therapy (CRT); GEMCAP = gemcitabine plus capecitabine; GEM-CRT = gemcitabine-based CRT.

Between December 24, 2009, and October 25, 2011, 114 patients were registered in the trial from 28 hospitals across the United Kingdom. All patients were followed until progression, death, or 12-month follow-up assessment. Seventy-four patients were eligible for randomization after 3 cycles of induction chemotherapy; 38 were allocated to receive Gem-CRT and 36 to receive Cap-CRT (Fig. 1) (3). HRQL data from patients who failed to proceed to randomization after induction were not included in this analysis because very few patients completed the questionnaire after disease progression.

### Questionnaire compliance and missing data

Questionnaire compliance was good throughout the study, baseline data being available for 34 (94%) of 36 patients receiving Cap-CRT and 35 (92%) of 38 patients receiving Gem-CRT (Table 2). Rates at the 39-week time point were reduced to 71% (Cap-CRT arm) and 66% (Gem-CRT arm). Importantly, fewer questionnaires were returned in the Gem-CRT arm during later time points due to higher rates of progression and death. Details and reasons for missing questionnaires are shown in Table 2. Table 3 suggests that those with missing questionnaires at later time points (particularly weeks 26 and 39) had worse overall survival than those who did complete questionnaires. No problems were reported regarding patients completing the questionnaires, but Table E3 (available online at www.redjournal.org) suggests that the scale of sexual satisfaction in the QLQ-PAN26 questionnaire was not completed as often as other scales. The reason for nonreturn was missing for more patients at week 23 than at other weeks. The week-23 assessment involved a clinic visit that was not part of standard care, and a number of centers did not return any CRFs for this time point, so we cannot ascertain for certain the reason for noncompletion, although it is likely to be administration error.

We received 305 questionnaires from all patients across all time points. Only 8 of the 32 HRQL scales had at least 1 missing item in more than 3% of the 305 questionnaires. Of those 8 scales, 7 had at least 1 missing item in less than 7% of the 305 questionnaires. The other scale, sexual dissatisfaction, had at least 1 missing item in 24% of the 305 questionnaires. Only those scales with more than at least half of the items completed could be imputed using the EORTC method, thus only 1 (sexual dissatisfaction) of the 32 scales had more than 3% of values imputed using the EORTC method. Data from the 52-week follow-up were omitted from further analyses due to the low return rate.

### HRQL during induction chemotherapy (weeks 0-17)

Baseline scores for functional scales were all greater than 64, similar to findings in other studies of pancreatic cancer. The range of possible scores is 0 to 100; our unpublished data show median scores for function scales of 90 to 100 in patients with symptomatic gallstones and in a sample of normal individuals (C. Johnson, unpublished data). Baseline scores for all symptom scores were below 50, except for future health concern (mean: 58.18). For comparison, patients with symptomatic gallstones score their pain at approximately 50 and normal individuals at <5 (C. Johnson, unpublished data).

**Fig. 2:**
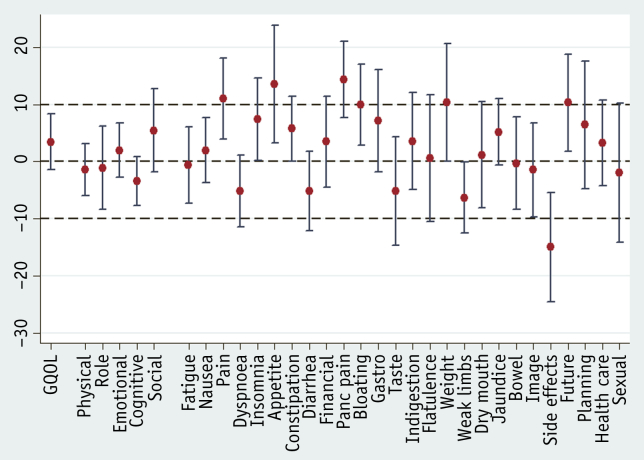
Changes in mean HRQL scores following induction chemotherapy (between week 0 and week 17) with 95% confidence intervals. Negative score indicates deterioration in both function and symptom scales. *Abbreviations:* GQOL = Global Quality of Life Scale; HRQL = health-related quality of life.

Figure 2 and Table E3 (available online at www.redjournal.org) show that, for all randomized patients, the mean changes in the majority of scales show improvement during induction chemotherapy with clinical significance achieved in the pain (−11.02; 95% confidence interval [CI]: −18.08 to −3.96), appetite loss (−13.56; 95% CI: −23.90 to −3.22), pancreatic pain (−14.32; 95% CI: −21.02 to −7.62), weight loss (−10.34; 95% CI: −20.62 to −0.06), and future health (−10.30; 95% CI: −18.78 to −1.83) scales. QLQ-PAN26 questions relating to side effects from treatment indicated significant deterioration (14.97; 95% CI: 5.38-24.55).

### HRQL during and after CRT

**Fig. 3:**
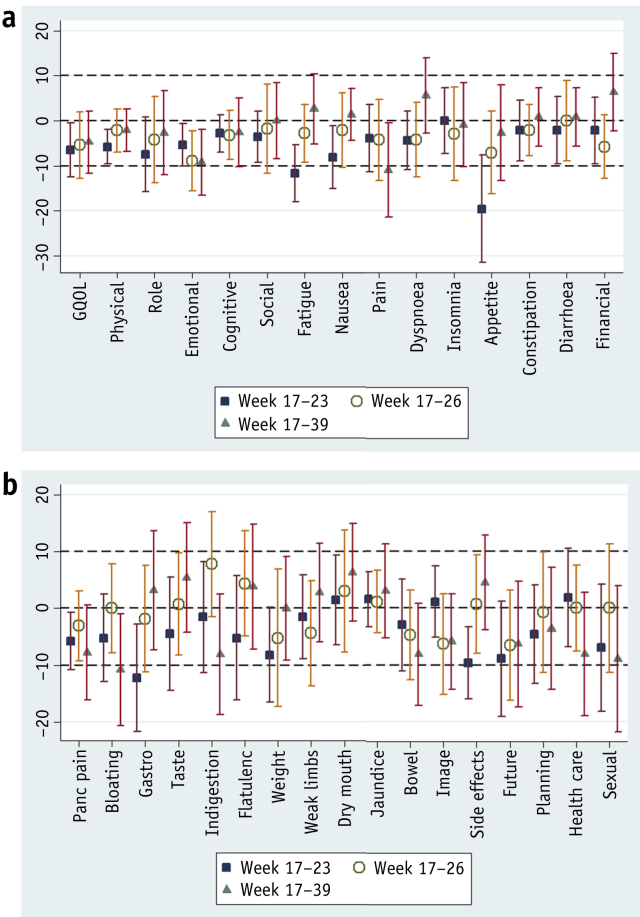
Changes in mean HRQL scores following chemoradiation (week 17 to later time points) with 95% confidence intervals. (a) QLQ-C30. (b) QLQ-PAN26. Negative score indicates deterioration in both functional and symptom scales.

Figure 3 and Table E4 (available online at www.redjournal.org) show the mean changes in scale scores between week 17 (start of CRT) and later time points of week 23 (at the end of CRT), week 26 (3 weeks post CRT), and week 39. Most scales deteriorated between the start (week 17) and end (week 23) of CRT. There was clinically significant deterioration including fatigue (11.70; 95% CI: 5.34-18.07), appetite loss (19.57; 95% CI: 7.65-31.48), and gastrointestinal symptoms (12.22; 95% CI: 2.83-21.61) and no clinically significant improvements over this period. However, there were no significant differences in mean scores between weeks 17 and 26, suggesting that recovery from the acute effects of CRT occurs within a 3-week period. At week 39 compared to week 17, there were clinically significant deteriorations including pain (10.96; 95% CI: 0.52-21.41) and bloating (10.81; 95% CI; 0.99-20.63).

### HRQL by trial arm

**Fig. 4:**
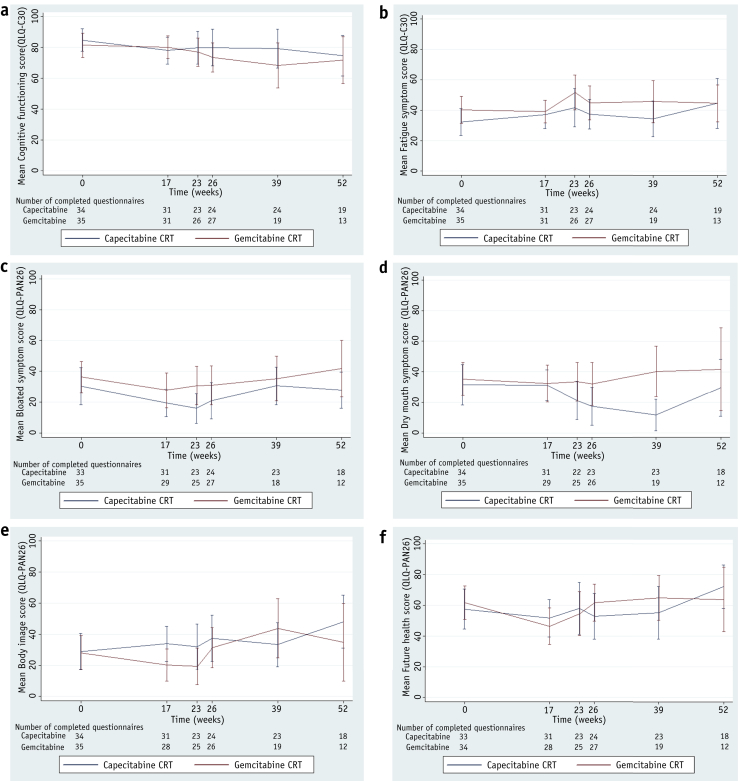
Changes in selected mean HRQL scores by treatment arm with 95% confidence intervals. (a) Cognitive functioning (high score indicates better QoL). (b) Fatigue (low score indicates better QoL). (c) Bloating (low score indicates better QoL). (d) Dry mouth (low score indicates better QoL). (e) Body image (low score indicates better QoL). (f) Future health concerns (low score indicates better QoL).

Table E5 (available online at www.redjournal.org) suggests that, due to chance, there were some imbalances in HRQL scale median scores at week 17 (the point of randomization) between arms. Thus, changes in score from week 17 and each subsequent time point were compared rather than absolute scores at each time point. Table E5 also shows the difference between trial arms in terms of change in scale scores between week 17 and later time points. The median change between week 17 and later time points was never worse in the Cap-CRT arm than in the Gem-CRT arm. Results of the Wilcoxon rank sum tests that compared differences between changes in score suggest little difference between arms, but where differences were found, each favored Cap-CRT. Between weeks 17 and 23, there were differences at the *P* level of <.05 between trial arms in the distribution of the change in the following scores: cognitive functioning (*P*=.036), fatigue (*P*=.046), bloating (*P*=.035), and dry mouth (*P*=.029). Between weeks 17 and 26, this was only significant for future health (*P*=.033). Between weeks 17 and 39, this was significant for cognitive functioning (*P*=.011), dry mouth (*P*=.001), and body image (*P*=.022). The only significant differences at the *P* level of <.01 was in dry mouth between weeks 17 and 39 (*P*=.001). Graphs of these selected domains are shown in Figure 4.

### Validation of the QLQ-PAN26 questionnaire during CRT

Cronbach's alpha was >.7 for all scales (implying good internal consistency), except for the jaundice scale (r=0.46). The jaundice scale has the following 2 questions: “have you had itching?” and “to what extent was your skin yellow?” The correlation between the scores for these 2 questions was low (Pearson correlation coefficient = 0.37).

Table E6 (available online at www.redjournal.org) shows the mean scores at week 23 in the group of patients who had any CTCAE grade 3 and 4 during CRT (primarily gastrointestinal and constitutional) and those who did not. Clinically significant differences were seen in 8 scales (primarily gastrointestinal and constitutional) with worse scores in the patients with more severe adverse events. There was a significantly worse mean score at 23 weeks in the “side effects of treatment” scale, comparing those who had a SAR during CRT and those who did not: 34.9 (n=44; 95% CI: 27.0-42.7) versus 50.0 (n=4; 95% CIs: −18.5 to 118.5), although the confidence intervals are wide due to the small numbers.

## Discussion

In the SCALOP trial there was improvement in most of the HRQL scales during induction chemotherapy. There was significant decline in a number of HRQL scales during CRT (fatigue, appetite loss, and gastrointestinal symptoms), but these recovered by 3 weeks after the end of CRT. We speculate that the clinically significant deterioration in pain and bloating scores at week 39 was likely to have been due to disease progression, either clinical or subclinical; however, as only 6 patients with documented progression had HRQL recorded at week 39, this conclusion is conjectural. The exploratory comparisons of differences in HRQL scores between trial arms rarely reached statistical significance, but where they did, they all favored Cap-CRT, providing support to our previously published data for the use of Cap-CRT rather than Gem-CRT.

How does SCALOP compare with other HRQL trials in LAPC? In the E4201 study, which randomized patients to single-agent gemcitabine- and gemcitabine-based CRT, decline in HRQL scores was noted during CRT, which returned to baseline levels within 9 weeks of completion of CRT (9). Despite a large difference in grade 4 toxicity between the arms, there were no statistically significant differences in median Functional Assessment of Cancer Therapy Hepatobiliary and Pancreatic subscale (FACT-Hep) scale score between the treatment arms. This may have been due to small patient numbers or to separation in time of the toxicity and HRQL assessment, so that the toxicity had resolved when HRQL was recorded. Short et al (10) reported HRQL using QLQ C30 and QLQ PAN26 questionnaires from a single-arm study, which included LAPC (n=41) and postoperative patients (n=22) receiving induction gemcitabine followed by 5-fluorouracil (5-FU)-based CRT (10). CRT improved local symptoms (pain scores and digestive symptoms), and the authors suggested that patients with local symptoms at baseline are most likely to benefit from CRT. Serrano et al (11) reported HRQL outcomes from a single-arm phase 2 trial of 2 cycles of neoadjuvant gemcitabine-oxaliplatin-based CRT (30 Gy in 15 fractions concurrent with first cycle) in patients with borderline resectable and resectable tumors (n=71) (11). This study reported a decline in global HRQL scores but an improvement in pancreatic pain at the end of neoadjuvant treatment. Long-term outcome in the unresected population was not reported due to low rates of questionnaire return. Contrary to these studies, SCALOP showed a temporary deterioration in local symptoms following CRT, although improvements in local symptoms were seen during induction chemotherapy.

The comparison of HRQL outcomes between LAPC patients treated with chemotherapy alone versus those receiving CRT remains an important but unanswered question. The clinical outcome from the LAP 07 trial, randomizing patients between chemotherapy alone and chemotherapy followed by induction chemotherapy, has been reported in abstract form only (12). That study showed no additional overall survival benefit for CRT over and above chemotherapy alone, calling into question the role of CRT in this disease. No HRQL data were collected in this trial.

This is the first study to validate the use of QLQ-PAN26 in patients receiving CRT, a treatment that was rarely used during its development. To our knowledge, the data presented here provide the most robust validation to date of the use of the QLQ-PAN26 in patients receiving CRT. Importantly, a range of scales and items showed deterioration between the start and end of CRT but with recovery by 3 weeks after the end of CRT. This corresponds well with expected side effects of CRT and demonstrates the ability of PAN26 to detect clinically relevant changes. Scales showed good correlation with nurse-reported adverse events and treatment-related toxicities. Finally, the scales also showed good internal consistency with the exception of the jaundice scale. This is not surprising, as all patients were free of jaundice during treatment.

Our study has several limitations. Patient numbers in each arm were relatively small, resulting in wide confidence intervals, and few of the observed differences achieved statistical significance. Also, comparing arms, results of the multiple tests conducted increased the probability of obtaining a *P* value of less than .05 by chance. Additionally, HRQL data from registered patients who did not proceed to randomization were not captured, restricting the longitudinal trends shown to a cohort of chemotherapy-selected patients with stable or responding disease and therefore better overall prognosis. Importantly, questionnaire return rates continued to decline through the study period, and it is likely those patients who did not respond to questionnaires during follow-up experienced a different HRQL profile. This may be a source of bias; however, data attrition is a significant problem in all studies of pancreatic cancer, largely due to the nature of the disease and patients' frequent rapidly declining health. Our data collection rate compares favorably with those of the E4201 trial and Serrano et al (11), in which HRQL questionnaire compliance was 40% at 9 months and 25% at 6 months, respectively (11).

## Conclusions

Despite these limitations, this study has confirmed the validity of the QLQ-PAN26 in patients receiving CRT. It provides detailed insight into HRQL following induction chemotherapy and consolidation CRT, which has not been previously described. These data will be useful when discussing therapeutic options in patients with LAPC and lend further support to the use of capecitabine rather than gemcitabine as the concomitant cytotoxic in this setting. Importantly, our data help to dispel any previously held anxieties and beliefs that CRT is a toxic treatment that will inevitably detract from HRQL in patients with limited life expectancy. The role of CRT in this disease remains controversial, and future trials in LAPC should incorporate HRQL end points.

## Figures and Tables

**Table 1:** QLQ scales, abbreviations, and imputations

Scale	Abbreviation	QLQ	Items in scale	Number of questionnaires with at least one item of the scale missing∗	Of those, number imputed by EORTC guidelines
Global	GQOL	C30	2	2	0
Functional					
Physical	Physical	C30	5	5	4
Role	Role	C30	2	2	0
Emotional	Emotional	C30	4	4	3
Cognitive	Cognitive	C30	2	0	0
Social	Social	C30	2	2	2
Symptoms					
Fatigue	Fatigue	C30	3	4	3
Nausea and vomiting	Nausea	C30	2	1	1
Pain	Pain	C30	2	5	5
Dyspnoea	Dyspnoea	C30	1	2	0
Insomnia	Insomnia	C30	1	2	0
Appetite loss	Appetite	C30	1	2	0
Constipation	Constipation	C30	1	1	0
Diarrhea	Diarrhoea	C30	1	3	0
Financial difficulties	Financial	C30	1	2	0
Pancreatic pain	Panc Pain	PAN26	4	14	8
Bloating	Bloating	PAN26	1	8	0
Gastrointestinal	Gastro	PAN26	2	8	1
Taste loss	Taste	PAN26	1	7	0
Indigestion	Indigestion	PAN26	1	11	0
Flatulence	Flatulence	PAN26	1	8	0
Weight	Weight	PAN26	1	7	0
Weak limbs	Weak limbs	PAN26	1	7	0
Dry mouth	Dry mouth	PAN26	1	9	0
Jaundice	Jaundice	PAN26	2	15	8
Altered bowel habit	Bowel	PAN26	2	13	6
Poor body image	Image	PAN26	2	11	3
Side effects of treatment	Side effects	PAN26	1	21	0
Future health concern	Future	PAN26	1	9	0
Forward planning limited	Planning	PAN26	1	9	0
Satisfaction with health care	Health care	PAN26	2	16	9
Sexual dissatisfaction	Sexual	PAN26	2	72	14

*Abbreviations:* C30 = General cancer module; EORTC = European Organization for Research and Treatment of Cancer; PAN26 = Pancreatic cancer module; QLQ = Quality of Life Questionnaire.

∗ Of the total of 305 questionnaires received from all patients across all time points.

**Table 2:** Questionnaire compliance and reasons for missing data by treatment group

	RT + capecitabine (n=36)												RT + gemcitabine (n=38)											
Baseline		17 weeks		23 weeks		26 weeks		39 weeks		52 weeks		Baseline		17 weeks		23 weeks		26 weeks		39 weeks		52 weeks	
n	%	n	%	n	%	n	%	n	%	n	%	n	%	n	%	n	%	n	%	n	%	n	%
Patients still alive	36		36		35		35		34		29		38		38		37		36		29		24	
CRFs returned	34	94	31	86	23	66	24	69	24	71	19	66	35	92	30	79	26	70	27	75	19	66	13	54
Reasons for nonreturn																								
Admin error	1	3	3	8	3	9	6	17	2	6	3	10	3	8	3	8	3	8	3	8	2	7	1	4
Patient declined	0	0	0	0	0	0	0	0	1	3	0	0	0	0	1	3	1	3	0	0	2	7	0	0
Patient too unwell	0	0	2	6	1	3	4	11	4	12	7	24	0	0	0	0	1	3	2	6	4	14	8	33
Unknown	1	3	0	0	8	23	1	3	3	9	0	0	0	0	4	11	6	16	4	11	2	7	2	8

*Abbreviations:* CRFs = Case report forms; RT = radiation therapy.

**Table 3:** Overall survival at each time point by questionnaire completion

Time point	Patients with missing questionnaire		Patients with questionnaire	
n	Overall survival (95% CIs)	n	Overall survival (95% CIs)
Week 17	13	14.6 (11.3-16.3)	61	15.8 (13.9-20.0)
Week 23	25	14.6 (10.3-16.3)	49	16.5 (14.0-21.5)
Week 26	23	12.7 (9.8-15.0)	51	19.1 (14.6-21.5)
Week 39	31	12.7 (9.5-14.0)	43	19.7 (15.7-23.1)

## Supplementary Data

Tables E3-E6
