# Supplementary material for: Health-Related Quality of Life in SCALOP, a Randomized Phase 2 Trial Comparing Chemoradiation Therapy Regimens in Locally Advanced Pancreatic Cancer
Source: Int J Radiat Oncol Biol Phys. 2015 Nov 15;93(4):810–8. doi: 10.1016/j.ijrobp.2015.08.026 (PMC4627359; doi:10.1016/j.ijrobp.2015.08.026)
Supplement: Tables E3-E6 [file mmc1.docx]

**Table 3. Mean difference in HRQL across all randomised patients between baseline and week 17 (induction chemotherapy)**

| **Scale** | **n** | **Wk 0 µ** | **Wk 17 µ** | **Diff** | **lCI** | **uCI** |
| --- | --- | --- | --- | --- | --- | --- |
| *Global* | 58 | 64.80 | 68.25 | 3.45 | -1.46 | 8.36 |
| *Functional* |  |  |  |  |  |  |
| Physical | 59 | 79.89 | 78.45 | -1.44 | -5.94 | 3.06 |
| Role | 59 | 70.90 | 69.77 | -1.13 | -8.43 | 6.17 |
| Emotional | 59 | 75.71 | 77.68 | 1.98 | -2.77 | 6.72 |
| Cognitive | 59 | 82.20 | 78.81 | -3.39 | -7.64 | 0.86 |
| Social | 58 | 65.52 | 70.98 | 5.46 | -1.85 | 12.77 |
| *Symptoms* |  |  |  |  |  |  |
| Fatigue | 59 | 37.29 | 37.85 | 0.56 | -6.12 | 7.25 |
| Nausea | 59 | 14.69 | 12.71 | -1.98 | -7.69 | 3.73 |
| Pain | 59 | 31.36 | 20.34 | **-11.02** | -18.08 | -3.96 |
| Dyspnoea | 58 | 13.22 | 18.39 | 5.17 | -1.14 | 11.49 |
| Insomnia | 59 | 34.46 | 27.12 | -7.34 | -14.57 | -0.12 |
| Appetite | 59 | 36.72 | 23.16 | **-13.56** | -23.90 | -3.22 |
| Constipation | 58 | 18.39 | 12.64 | -5.75 | -11.47 | -0.03 |
| Diarrhoea | 58 | 20.11 | 25.29 | 5.17 | -1.75 | 12.10 |
| Financial | 57 | 21.05 | 17.54 | -3.51 | -11.47 | 4.45 |
| Panc Pain | 58 | 36.83 | 22.51 | **-14.32** | -21.02 | -7.62 |
| Bloating | 57 | 32.75 | 22.81 | -9.94 | -17.03 | -2.86 |
| Gastro | 58 | 35.92 | 28.74 | -7.18 | -16.14 | 1.78 |
| Taste | 58 | 27.01 | 32.18 | 5.17 | -4.37 | 14.72 |
| Indigestion | 56 | 25.60 | 22.02 | -3.57 | -12.03 | 4.88 |
| Flatulence | 58 | 48.85 | 48.28 | -0.57 | -11.65 | 10.50 |
| Weight | 58 | 40.80 | 30.46 | **-10.34** | -20.62 | -0.06 |
| Weak limbs | 58 | 27.01 | 33.33 | 6.32 | 0.08 | 12.56 |
| Dry mouth | 58 | 32.18 | 31.03 | -1.15 | -10.43 | 8.13 |
| Jaundice | 55 | 16.97 | 11.82 | -5.15 | -10.96 | 0.66 |
| Bowel | 58 | 34.48 | 34.77 | 0.29 | -7.78 | 8.35 |
| Image | 57 | 26.02 | 27.49 | 1.46 | -6.78 | 9.70 |
| Side effects | 49 | 15.65 | 30.61 | **14.97** | 5.38 | 24.55 |
| Future | 55 | 58.18 | 47.88 | **-10.30** | -18.78 | -1.83 |
| Planning | 57 | 40.35 | 33.92 | -6.43 | -17.58 | 4.71 |
| Healthcare | 57 | 85.96 | 89.18 | 3.22 | -4.28 | 10.71 |
| Sexual | 43 | 50.00 | 51.94 | 1.94 | -10.23 | 14.11 |

Abbreviations: µ=mean; lCI=lower 95% confidence interval; uCI=upper 95% confidence interval; Diff= difference between means

NB High scores in function scales (and SatHC) represent better HRQLand higher scores in symptom scales represent worse HRQL. Negative differences in function scales represent HRQLworsening whereas negative differences in symptom scores represents HRQL improvement. Clinically significant differences are highlighted in bold.

**Table 4. Mean difference in HRQL across all randomised patients between week 17 and later timepoints**

|  | **Week 23-17** | | | | | | **Week 26-17** | | | | | | **Week 39-17** | | | | | |
| --- | --- | --- | --- | --- | --- | --- | --- | --- | --- | --- | --- | --- | --- | --- | --- | --- | --- | --- |
| **Scale** | **n** | **Wk 17 µ** | **Wk 23 µ** | **Diff** | **lCI** | **uCI** | **n** | **Wk 17 µ** | **Wk 26 µ** | **Diff** | **lCI** | **uCI** | **n** | **Wk 17 µ** | **Wk 39 µ** | **Diff** | **lCI** | **uCI** |
| *Functional* |  |  |  |  |  |  |  |  |  |  |  |  |  |  |  |  |  |  |
| GQOL | 46 | 68.84 | 62.32 | -6.52 | -12.49 | -0.55 | 46 | 67.39 | 61.96 | -5.43 | -12.76 | 1.89 | 37 | 66.44 | 61.71 | -4.73 | -11.62 | 2.16 |
| Physical | 47 | 77.84 | 72.06 | -5.78 | -9.65 | -1.91 | 47 | 78.55 | 76.35 | -2.20 | -6.95 | 2.55 | 37 | 76.76 | 74.59 | -2.16 | -6.88 | 2.55 |
| Role | 47 | 70.92 | 63.48 | -7.45 | -15.66 | 0.77 | 47 | 69.15 | 64.89 | -4.26 | -13.85 | 5.34 | 37 | 66.22 | 63.51 | -2.70 | -12.01 | 6.61 |
| Emotional | 47 | 78.55 | 73.17 | -5.38 | -10.08 | -0.68 | 47 | 76.95 | 68.09 | -8.87 | -15.51 | -2.22 | 38 | 77.85 | 68.64 | -9.21 | -16.49 | -1.93 |
| Cognitive | 47 | 80.50 | 77.66 | -2.84 | -6.96 | 1.29 | 47 | 79.08 | 75.89 | -3.19 | -8.65 | 2.27 | 38 | 76.75 | 74.12 | -2.63 | -10.22 | 4.96 |
| Social | 46 | 69.20 | 65.58 | -3.62 | -9.33 | 2.08 | 46 | 66.67 | 64.86 | -1.81 | -11.72 | 8.10 | 37 | 63.06 | 63.06 | 0.00 | -8.49 | 8.49 |
| *Symptoms* |  |  |  |  |  |  |  |  |  |  |  |  |  |  |  |  |  |  |
| Fatigue | 47 | 35.22 | 46.93 | **11.70** | 5.34 | 18.07 | 47 | 38.06 | 40.90 | 2.84 | -3.53 | 9.20 | 38 | 40.35 | 37.72 | -2.63 | -10.39 | 5.12 |
| Nausea | 47 | 12.06 | 20.21 | 8.16 | 1.17 | 15.14 | 47 | 13.12 | 15.25 | 2.13 | -6.07 | 10.33 | 38 | 11.84 | 10.53 | -1.32 | -7.07 | 4.43 |
| Pain | 47 | 20.92 | 24.82 | 3.90 | -3.54 | 11.34 | 47 | 21.99 | 26.24 | 4.26 | -4.73 | 13.24 | 38 | 20.61 | 31.58 | **10.96** | 0.52 | 21.41 |
| Dyspnoea | 46 | 19.57 | 23.91 | 4.35 | -2.12 | 10.82 | 47 | 20.57 | 24.82 | 4.26 | -4.06 | 12.57 | 36 | 22.22 | 16.67 | -5.56 | -13.87 | 2.75 |
| Insomnia | 47 | 26.24 | 26.24 | 0.00 | -7.36 | 7.36 | 46 | 28.26 | 31.16 | 2.90 | -7.50 | 13.30 | 37 | 27.93 | 28.83 | 0.90 | -8.36 | 10.16 |
| Appetite | 46 | 19.57 | 39.13 | **19.57** | 7.65 | 31.48 | 47 | 23.40 | 30.50 | 7.09 | -2.02 | 16.20 | 37 | 23.42 | 26.13 | 2.70 | -7.90 | 13.30 |
| Constipation | 46 | 14.49 | 16.67 | 2.17 | -4.56 | 8.90 | 46 | 15.94 | 18.12 | 2.17 | -3.50 | 7.85 | 38 | 15.79 | 14.91 | -0.88 | -7.37 | 5.61 |
| Diarrhoea | 47 | 19.86 | 21.99 | 2.13 | -5.34 | 9.60 | 46 | 23.19 | 23.19 | 0.00 | -8.85 | 8.85 | 38 | 21.93 | 21.05 | -0.88 | -7.37 | 5.61 |
| Financial | 46 | 20.29 | 22.46 | 2.17 | -5.18 | 9.52 | 46 | 18.12 | 23.91 | 5.80 | -1.22 | 12.81 | 37 | 20.72 | 14.41 | -6.31 | -14.93 | 2.32 |
| Panc Pain | 45 | 21.85 | 27.59 | 5.74 | 0.72 | 10.77 | 46 | 24.46 | 27.54 | 3.08 | -3.03 | 9.19 | 37 | 22.45 | 30.18 | 7.73 | -0.55 | 16.02 |
| Bloating | 45 | 19.26 | 24.44 | 5.19 | -2.50 | 12.87 | 46 | 25.36 | 25.36 | 0.00 | -7.81 | 7.81 | 37 | 20.72 | 31.53 | **10.81** | 0.99 | 20.63 |
| Gastro | 45 | 25.56 | 37.78 | **12.22** | 2.83 | 21.61 | 46 | 30.80 | 32.61 | 1.81 | -7.59 | 11.22 | 37 | 27.03 | 23.87 | -3.15 | -13.62 | 7.31 |
| Taste | 45 | 31.11 | 35.56 | 4.44 | -5.48 | 14.37 | 46 | 36.96 | 36.23 | -0.72 | -9.70 | 8.25 | 37 | 29.73 | 24.32 | -5.41 | -15.04 | 4.22 |
| Indigestion | 44 | 20.45 | 21.97 | 1.52 | -8.25 | 11.28 | 43 | 25.58 | 17.83 | -7.75 | -16.94 | 1.43 | 37 | 22.52 | 30.63 | 8.11 | -2.50 | 18.72 |
| Flatulence | 45 | 43.70 | 48.89 | 5.19 | -5.69 | 16.06 | 46 | 47.10 | 42.75 | -4.35 | -13.59 | 4.89 | 35 | 53.33 | 49.52 | -3.81 | -14.84 | 7.22 |
| Weight | 45 | 30.37 | 38.52 | 8.15 | -0.16 | 16.46 | 45 | 29.63 | 34.81 | 5.19 | -6.88 | 17.25 | 37 | 31.53 | 31.53 | 0.00 | -9.07 | 9.07 |
| Weak limbs | 45 | 30.37 | 31.85 | 1.48 | -5.90 | 8.86 | 46 | 31.16 | 35.51 | 4.35 | -4.89 | 13.59 | 36 | 33.33 | 30.56 | -2.78 | -11.46 | 5.91 |
| Dry mouth | 44 | 28.79 | 27.27 | -1.52 | -9.38 | 6.35 | 44 | 29.55 | 26.52 | -3.03 | -13.70 | 7.64 | 37 | 28.83 | 22.52 | -6.31 | -14.93 | 2.32 |
| Jaundice | 43 | 10.08 | 8.53 | -1.55 | -6.41 | 3.30 | 44 | 10.23 | 9.09 | -1.14 | -6.64 | 4.37 | 33 | 13.64 | 10.61 | -3.03 | -11.32 | 5.26 |
| Bowel | 45 | 31.11 | 34.07 | 2.96 | -5.12 | 11.04 | 46 | 35.51 | 40.22 | 4.71 | -3.21 | 12.63 | 37 | 37.39 | 45.50 | 8.11 | -0.84 | 17.05 |
| Image | 45 | 25.93 | 24.81 | -1.11 | -7.37 | 5.15 | 45 | 27.78 | 34.07 | 6.30 | -2.53 | 15.12 | 37 | 28.83 | 34.68 | 5.86 | -2.55 | 14.26 |
| Side effects | 45 | 28.15 | 37.78 | 9.63 | 3.36 | 15.90 | 45 | 34.07 | 33.33 | -0.74 | -9.41 | 7.93 | 37 | 35.14 | 30.63 | -4.50 | -12.86 | 3.85 |
| Future | 45 | 48.89 | 57.78 | 8.89 | -1.22 | 18.99 | 46 | 51.45 | 57.97 | 6.52 | -3.18 | 16.22 | 37 | 50.45 | 56.76 | 6.31 | -4.76 | 17.37 |
| Planning | 44 | 34.09 | 38.64 | 4.55 | -4.08 | 13.18 | 46 | 37.68 | 38.41 | 0.72 | -9.81 | 11.26 | 37 | 38.74 | 42.34 | 3.60 | -7.13 | 14.34 |
| Healthcare | 45 | 89.63 | 91.48 | 1.85 | -6.77 | 10.47 | 46 | 92.03 | 92.03 | 0.00 | -7.60 | 7.60 | 37 | 92.34 | 84.23 | -8.11 | -18.96 | 2.74 |
| Sexual | 36 | 53.70 | 60.65 | 6.94 | -4.20 | 18.09 | 38 | 53.95 | 53.95 | 0.00 | -11.32 | 11.32 | 30 | 49.44 | 58.33 | 8.89 | -3.95 | 21.73 |

Abbreviations: µ=mean; lCI=lower 95% confidence interval; uCI=upper 95% confidence interval; Diff= difference between means; scale abbreviations as shown in Table 1.

NB High scores in functioning scales (and SatHC) represent better HRQL and higher scores in symptom scales represent worse HRQL. Negative differences in functioning scales (and SatHC) represent HRQLworsening whereas negative differences in symptom scores represent HRQLimprovement. Clinically significant differences are highlighted in bold.

**Table 5. Difference in HRQL between week 17 and later timepoints by trial arm**

|  | Week 23-Week 17 | | | | | | | | Week 26-Week 17 | | | | | | Week 39-Week 17 | | | | | |
| --- | --- | --- | --- | --- | --- | --- | --- | --- | --- | --- | --- | --- | --- | --- | --- | --- | --- | --- | --- | --- |
|  | Capecitabine | | | Gemcitabine | | |  |  | Capecitabine | | Gemcitabine | |  |  | Capecitabine | | Gemcitabine | |  |  |
| Scale | Median score week 17 | n | Median difference in score (range) | Median score week 17 | n | Median difference in score (range) | z | p | n | Median difference in score (range) | n | Median difference in score (range) | z | p | n | Median difference in score (range) | n | Median difference in score (range) | z | p |
| *Func.* |  |  |  |  |  |  |  |  |  |  |  |  |  |  |  |  |  |  |  |  |
| GQOL | 66.7 | 23 | 0.0  (-33.3,41.7) | 66.7 | 23 | -16.7  (50.0,33.3) | 1.70 | 0.090 | 23 | 0.0  (-50.0,50.0) | 23 | 0.0  (-83.3,33.3) | 0.57 | 0.571 | 23 | 0.0  (-41.7,33.3) | 14 | -8.3  (-58.3,33.3) | 1.21 | 0.227 |
| Physical | 86.7 | 23 | 0.0  (-40.0,26.7) | 73.3 | 24 | -10.0  (-33.3,20.0) | 1.58 | 0.114 | 23 | 0.0  (-33.3,26.7) | 24 | 0.0  (-66.7,26.7) | -0.24 | 0.808 | 22 | 0.0  (-13.3,26.7) | 15 | -6.7  (-40.0,13.3) | 1.28 | 0.199 |
| Role | 83.3 | 23 | 0.0  (-50.0,66.7) | 66.7 | 24 | -16.7  (-66.7,66.7) | 1.11 | 0.268 | 23 | 0.0  (-50.0,66.7) | 24 | -16.7  (-100.0,66.7) | 1.13 | 0.259 | 22 | 0.0  (-50.0,50.0) | 15 | 0.0  (-66.7,33.3) | 1.22 | 0.223 |
| Emotional | 83.3 | 23 | 0.0  (-58.3,16.7) | 75.0 | 24 | 0.0  (-58.3,25.0) | -0.17 | 0.869 | 23 | 0.0  (-33.3,25.0) | 24 | -8.3  (-83.3,41.7) | 1.67 | 0.094 | 23 | -8.3  (-50.0,16.7) | 15 | -8.3  (-75.0,50.0) | 0.53 | 0.598 |
| Cognitive | 83.3 | 23 | 0.0  (-16.7,16.7) | 83.3 | 24 | 0.0  (-33.3,33.3) | 2.10 | **0.036** | 23 | 0.0  (-33.3,33.3) | 24 | 0.0  (-66.7,16.7) | 1.19 | 0.233 | 23 | 0.0  (-66.7,33.3) | 15 | -16.7  (-50.0,33.3) | 2.54 | **0.011** |
| Social | 83.3 | 23 | 0.0  (-66.7,50.0) | 66.7 | 23 | 0.0  (-33.3,33.3) | 0.19 | 0.848 | 23 | 0.0  (-66.7,66.7) | 23 | 0.0  (-100.0,33.3) | 0.80 | 0.422 | 23 | 0.0  (-66.7,50.0) | 14 | 0.0  (-66.7,33.3) | -0.02 | 0.987 |
| *Symp.* |  |  |  |  |  |  |  |  |  |  |  |  |  |  |  |  |  |  |  |  |
| Fatigue | 33.3 | 23 | 11.1  (-33.3,44.4) | 33.3 | 24 | 16.7  (-33.3,55.6) | -1.99 | **0.046** | 23 | 0.0  (-44.4,33.3) | 24 | 0.0  (-33.3,77.8) | -0.45 | 0.652 | 23 | -11.1  (-66.7,33.3) | 15 | -11.1  (-22.2,44.4) | -1.47 | 0.142 |
| Nausea | 0.0 | 23 | 0.0  (-33.3,50.0) | 16.7 | 24 | 8.3  (-50.0,83.3) | -1.00 | 0.320 | 23 | 0.0  (-33.3,50.0) | 24 | 0.0  (-66.7,100.0) | 0.38 | 0.700 | 23 | 0.0  (-33.3,50.0) | 15 | 0.0  (-33.3,0.0) | 1.83 | 0.067 |
| Pain | 16.7 | 23 | 0.0  (-33.3,33.3) | 16.7 | 24 | 0.0  (-66.7,83.3) | -1.36 | 0.174 | 23 | 0.0  (-50.0,66.7) | 24 | 0.0  (-66.7,100.0) | -0.79 | 0.431 | 23 | 0.0  (-66.7,66.7) | 15 | 16.7  (-50.0,66.7) | -0.61 | 0.545 |
| Dyspnoea | 0.0 | 22 | 0.0  (-66.7,33.3) | 33.3 | 24 | 0.0  (-33.3,66.7) | -1.52 | 0.129 | 23 | 0.0  (-66.7,66.7) | 24 | 0.0  (-33.3,66.7) | -1.19 | 0.234 | 21 | 0.0  (-66.7,33.3) | 15 | 0.0  (-33.3,66.7) | -1.38 | 0.167 |
| Insomnia | 33.3 | 23 | 0.0  (-66.7,33.3) | 0.0 | 24 | 0.0  (-33.3,66.7) | -0.48 | 0.635 | 22 | 0.0  (-66.7,33.3) | 24 | 0.0  (-66.7,100.0) | 0.12 | 0.907 | 22 | 0.0  (-66.7,66.7) | 15 | 0.0  (-33.3,66.7) | -0.96 | 0.335 |
| Appetite | 0.0 | 23 | 0.0  (-66.7,100.0) | 33.3 | 23 | 33.3  (-66.7,100.0) | -1.75 | 0.081 | 23 | 0.0  (-66.7,100.0) | 24 | 0.0  (-33.3,66.7) | -0.80 | 0.421 | 22 | 0.0  (-33.3,100.0) | 15 | 0.0  (-66.7,66.7) | 0.12 | 0.904 |
| Constipation | 0.0 | 22 | 0.0  (-33.3,66.7) | 0.0 | 24 | 0.0  (-33.3,33.3) | -0.64 | 0.525 | 22 | 0.0  (-33.3,33.3) | 24 | 0.0  (-33.3,33.3) | -0.65 | 0.516 | 23 | 0.0  (-66.7,33.3) | 15 | 0.0  (-33.3,33.3) | -0.66 | 0.511 |
| Diarrhoea | 0.0 | 23 | 0.0  (-33.3,33.3) | 33.3 | 24 | 0.0  (-66.7,66.7) | 0.42 | 0.678 | 22 | 0.0  (-33.3,66.7) | 24 | 0.0  (-66.7,66.7) | 0.03 | 0.980 | 23 | 0.0  (-33.3,33.3) | 15 | 0.0  (-33.3,33.3) | -0.21 | 0.831 |
| Financial | 0.0 | 23 | 0.0  (-33.3,33.3) | 0.0 | 23 | 0.0  (-33.3,100.0) | -1.12 | 0.262 | 23 | 0.0  (-33.3,66.7) | 23 | 0.0  (-33.3,66.7) | -0.87 | 0.383 | 23 | 0.0  (-66.7,66.7) | 14 | 0.0  (-66.7,33.3) | 0.51 | 0.613 |
| Panc Pain | 8.3 | 23 | 0.0  (-33.3,33.3) | 25.0 | 22 | 8.3  (-16.7,50.0) | -0.47 | 0.640 | 23 | 0.0  (-41.7,50.0) | 23 | 0.0  (-33.3,50.0) | 0.13 | 0.894 | 22 | 8.3  (-50.0,58.3) | 15 | 0.0  (-33.3,75.0) | 0.19 | 0.851 |
| Bloating | 0.0 | 23 | 0.0  (-33.3,33.3) | 33.3 | 22 | 0.0  (-33.3,100.0) | -2.11 | **0.035** | 23 | 0.0  (-33.3,33.3) | 23 | 0.0  (-66.7,66.7) | -0.62 | 0.536 | 22 | 0.0  (-66.7,100.0) | 15 | 0.0  (-33.3,33.3) | 0.42 | 0.674 |
| Gastro | 16.7 | 23 | 0.0  (-16.7,100.0) | 33.3 | 22 | 8.3 (-50.0,66.7) | -0.36 | 0.718 | 23 | 0.0  (-66.7,66.7) | 23 | 0.0  (-50.0,100.0) | 0.35 | 0.726 | 22 | 0.0  (-66.7,66.7) | 15 | -16.7  (-50.0,50.0) | 1.66 | 0.097 |
| Taste | 33.3 | 23 | 0.0  (-100.0,66.7) | 33.3 | 22 | 0.0 (-33.3,100.0) | -0.91 | 0.361 | 23 | 0.0  (-66.7,66.7) | 23 | 0.0  (-66.7,66.7) | -0.60 | 0.546 | 22 | 0.0  (-66.7,33.3) | 15 | 0.0  (-66.7,33.3) | -0.34 | 0.732 |
| Indigestion | 0.0 | 22 | 0.0  (-33.3,100.0) | 33.3 | 22 | 0.0  (-33.3,66.7) | 0.11 | 0.915 | 21 | 0.0  (-100.0,66.7) | 22 | 0.0  (-66.7,33.3) | 1.17 | 0.244 | 22 | 0.0  (-66.7,100.0) | 15 | 0.0 (0.0,33.3) | -0.45 | 0.651 |
| Flatulence | 33.3 | 23 | 0.0  (-33.3,100.0) | 33.3 | 22 | 0.0  (-100,100) | -0.01 | 0.990 | 23 | 0.0  (-100.0,66.7) | 23 | 0.0  (-66.7,33.3) | 0.30 | 0.761 | 21 | 0.0  (-66.7,100.0) | 14 | 0.0  (-33.3,33.3) | 0.19 | 0.852 |
| Weight | 33.3 | 23 | 0.0  (-33.3,66.7) | 33.3 | 22 | 0.0  (-66.7,66.7) | -1.02 | 0.310 | 22 | 0.0  (-66.7,66.7) | 23 | 0.0  (-100,100) | -0.73 | 0.467 | 22 | 0.0  (-33.3,66.7) | 15 | 0.0  (-66.7,66.7) | 0.34 | 0.738 |
| Weak limbs | 33.3 | 23 | 0.0  (-66.7,33.3) | 33.3 | 22 | 0.0  (-33.3,66.7) | -1.76 | 0.078 | 23 | 0.0  (-66.7,33.3) | 23 | 0.0  (-33.3,100.0) | -1.37 | 0.169 | 21 | 0.0  (-33.3,33.3) | 15 | 0.0  (-33.3,66.7) | -1.62 | 0.104 |
| Dry mouth | 33.3 | 22 | 0.0  (-66.7,33.3) | 33.3 | 22 | 0.0  (-66.7,33.3) | -2.18 | **0.029** | 22 | 0.0  (-66.7,66.7) | 22 | 0.0  (-66.7,100.0) | -1.23 | 0.220 | 22 | 0.0  (-66.7,0.0) | 15 | 0.0  (-33.3,33.3) | -3.44 | **0.001** |
| Jaundice | 0.0 | 22 | 0.0  (-33.3,50.0) | 0.0 | 21 | 0.0  (-50.0,33.3) | 0.62 | 0.536 | 22 | 0.0  (-50.0,66.7) | 22 | 0.0  (-33.3,16.7) | -0.05 | 0.956 | 19 | 0.0  (-66.7,50.0) | 14 | 0.0  (-66.7,33.3) | -0.12 | 0.907 |
| Bowel | 33.3 | 23 | 0.0  (-33.3,66.7) | 50.0 | 22 | 0.0  (-50.0,66.7) | 1.02 | 0.306 | 23 | 0.0  (-50.0,66.7) | 23 | 0.0  (-50.0,50.0) | -0.45 | 0.652 | 22 | 0.0  (-50.0,66.7) | 15 | 0.0  (-33.3,50.0) | 0.00 | 1.000 |
| Image | 33.3 | 23 | 0.0  (-33.3,50.0) | 0.0 | 22 | 0.0  (-66.7,33.3) | 0.51 | 0.608 | 23 | 0.0  (-50.0,50.0) | 22 | 0.0  (-50.0,100.0) | -0.28 | 0.778 | 22 | 0.0  (-50.0,33.3) | 15 | 0.0  (-16.7,66.7) | -2.29 | **0.022** |
| Side effects | 33.3 | 23 | 0.0  (-33.3,33.3) | 33.3 | 22 | 0.0 (-33.3,66.7) | -0.55 | 0.584 | 23 | 0.0  (-66.7,33.3) | 22 | 0.0  (-33.3,100.0) | -0.15 | 0.883 | 22 | 0.0  (-33.3,33.3) | 15 | 0.0  (-33.3,33.3) | -0.02 | 0.987 |
| Future | 66.7 | 23 | 0.0  (-33.3,66.7) | 33.3 | 22 | 0.0  (-66.7,100.0) | -0.32 | 0.747 | 23 | 0.0  (-33.3,66.7) | 23 | 0.0  (-33.3,100.0) | -2.13 | **0.033** | 22 | 0.0  (-33.3,100.0) | 15 | 0.0  (-66.7,66.7) | -0.52 | 0.600 |
| Planning | 33.3 | 23 | 0.0  (-33.3,66.7) | 33.3 | 21 | 0.0  (-33.3,66.7) | 0.20 | 0.841 | 23 | 0.0  (-100.0,66.7) | 23 | 0.0  (-66.7,66.7) | -0.85 | 0.396 | 22 | 0.0  (-66.7,100.0) | 15 | 0.0  (-33.3,66.7) | -0.80 | 0.426 |
| Healthcare | 100.0 | 23 | 0.0  (-100,100) | 100.0 | 22 | 0.0  (-16.7,83.3) | -0.76 | 0.449 | 23 | 0.0 (-100.0,100.0) | 23 | 0.0  (-33.3,50.0) | -0.25 | 0.803 | 22 | 0.0  (-100.0,16.7) | 15 | 0.0  (-50.0,50.0) | -1.91 | 0.056 |
| Sexual | 41.7 | 20 | 0.0  (-66.7,66.7) | 66.7 | 16 | 0.0  (-100.0,83.3) | -0.07 | 0.941 | 20 | 0.0 (0.0,66.7) | 18 | 0.0  (-100.0,83.3) | 1.24 | 0.213 | 17 | 0.0  (-33.3,33.3) | 13 | 0.0  (-100.0,83.3) | -0.55 | 0.583 |

NB: Negative differences in functioning scales (and SatHC) represent HRQL worsening whereas negative differences in symptom scores represents HRQL improvement. Abbreviations as shown in Table 1. P values < 0.05 are in bold.

**Table 6. Mean week 23 QLQ-PAN26 scale scores by those with and without any CTCAE grade 3 or 4 adverse events during CRT**

| **PAN 26 scale** | **No grade 3 or 4 adverse events during CRT** | | | | **Any Grade 3 or 4 adverse events during CRT** | | | |
| --- | --- | --- | --- | --- | --- | --- | --- | --- |
|  | **n** | **Mean** | **Lower 95% CI** | **Upper 95% CI** | **n** | **Mean** | **Lower 95% CI** | **Upper 95% CI** |
| Pancreatic pain* | 38 | 23.90 | 16.93 | 30.88 | 10 | 36.67 | 18.20 | 55.14 |
| Bloating* | 38 | 20.18 | 11.52 | 28.83 | 10 | 36.67 | 15.79 | 57.55 |
| Gastrointestinal* | 38 | 32.89 | 21.40 | 44.39 | 10 | 58.33 | 35.68 | 80.99 |
| Taste loss | 38 | 34.21 | 24.19 | 44.24 | 10 | 40.00 | 10.69 | 69.31 |
| Indigestion | 38 | 21.05 | 11.38 | 30.73 | 10 | 23.33 | 0.71 | 45.95 |
| Flatulence | 38 | 45.61 | 34.98 | 56.25 | 10 | 50.00 | 26.83 | 73.17 |
| Weight* | 38 | 31.58 | 20.20 | 42.96 | 10 | 63.33 | 32.65 | 94.01 |
| Weak limbs* | 38 | 26.32 | 17.43 | 35.20 | 10 | 53.33 | 30.30 | 76.37 |
| Dry mouth | 37 | 28.83 | 18.63 | 39.03 | 10 | 23.33 | 3.70 | 42.96 |
| Jaundice | 38 | 7.89 | 2.84 | 12.95 | 10 | 8.33 | 2.05 | 14.62 |
| Altered bowel habit* | 38 | 28.51 | 18.41 | 38.61 | 10 | 58.33 | 33.67 | 82.99 |
| Poor body image | 38 | 25.44 | 14.80 | 36.07 | 10 | 25.00 | 3.05 | 46.95 |
| Side effects of treatment | 38 | 35.96 | 27.37 | 44.56 | 10 | 36.67 | 12.95 | 60.38 |
| Future health concern* | 38 | 51.75 | 39.89 | 63.62 | 10 | 73.33 | 48.71 | 97.96 |
| Forward planning limited* | 37 | 33.33 | 21.91 | 44.75 | 10 | 56.67 | 37.04 | 76.30 |
| Satisfaction with healthcare | 38 | 92.11 | 85.52 | 98.69 | 10 | 91.67 | 81.53 | 101.80 |
| Sexual dissatisfaction | 31 | 59.14 | 43.45 | 74.83 | 7 | 66.67 | 32.20 | 101.13 |

 *indicates a difference in means of more than 10 points
